# Artificial Intelligence-Based Diagnostic Support System for Functional Dyspepsia Based on Brain Activity and Food Preference

**DOI:** 10.7759/cureus.49877

**Published:** 2023-12-03

**Authors:** Ryo Katsumata, Takayuki Hosokawa, Tomoari Kamada

**Affiliations:** 1 Department of Health Care Medicine, Kawasaki Medical School General Medical Center, Okayama, JPN; 2 Department of Orthoptics, Faculty of Rehabilitation, Kawasaki University of Medical Welfare, Kurashiki, JPN

**Keywords:** artificial intelligence, prefrontal cortex, near-infrared spectroscopy, ibs (irritable bowel syndrome), functional dyspepsia

## Abstract

Background and aim

Disorders of gut-brain interaction (DGBI) are disorders where no organic clinical abnormalities are detected such as functional dyspepsia (FD) and irritable bowel syndrome (IBS). The brain activity of individuals with FD and IBS differs from that of healthy controls. Artificial intelligence can distinguish healthy controls from individuals with DGBI using several biomarkers. This study aimed to establish an artificial intelligence-based diagnostic support system using food preferences and brain activity in patients with DGBI.

Methods

ROME IV criteria were used to diagnose patients with FD and IBS. Their food preference was scored using a visual analog scale, and brain activity in the prefrontal cortex was investigated using functional near-infrared spectroscopy (fNIRS). The diagnostic model was developed based on the brain activity and visual analog scale scores for food using an artificial neural network model.

Results

Forty-one participants, including 25 patients with DGBI were enrolled in the study. The accuracy of the artificial intelligence-based diagnostic model using an artificial neural network in differentiating between healthy controls and patients with DGBI and between healthy controls and those with FD were 72.3% and 77.1%, respectively.

Conclusions

The artificial intelligence-based diagnostic model using brain activity and preference to food images showed sufficiently high accuracy in distinguishing patients with DGBI from healthy controls, and those with FD from healthy controls. Therefore, the fNIRS system provides objective evidence for diagnosing DGBI.

## Introduction

Disorders of gut-brain interaction (DGBI), such as functional dyspepsia (FD) and irritable bowel syndrome (IBS), are diagnosed when clinicians cannot detect any organic abnormalities through clinical examination in ordinary clinical settings [1-3]. Moreover, diagnosis using objective parameters is challenging since FD and IBS are associated with several pathophysiological factors, including impaired disrupted gut-brain interaction, disabled motility, and the impact of food [3].

Fatty food intake reportedly induced gastrointestinal (GI) symptoms in patients with FD and those with IBS [4,5]. Additionally, our previous study showed that preference scores for fatty food images were significantly lower in patients with DGBI, particularly those with FD [6]. Regarding brain activity in patients with DGBIs, previous studies have reported alterations in several brain regions in patients with FD and those with IBS when compared to healthy controls [7,8]. Our previous study also showed altered prefrontal cortex (PFC) activity in response to food images in patients with DGBI, particularly those with FD [6]. We adopted functional near-infrared spectroscopy (fNIRS) to evaluate brain activity in PFC because of its non-invasiveness, portability, and ability to assess real-time brain activity [9]. Furthermore, data obtained from fNIRS have been proven to be sufficiently correlated with those of functional magnetic resonance imaging (fMRI) [10,11].

Artificial intelligence (AI) has been used to support the imaging diagnosis of GI disorders, including gastric and colon cancers using endoscopic findings and hepatic cancer using computed tomography imaging [12,13]. There are several AI-based diagnostic systems for DGBIs using gut-microbiome, pulse signal, bowel sound features, and endoscopic findings [14,15], and in terms of food, the efficacy of an AI-based personalized diet has been confirmed [16]. However, a model that uses food preference and brain activity in response to food images has not yet been established.

Therefore, this pilot study aimed to establish a diagnostic support system using an artificial neural network that employs food preference and brain activity responses to food images in patients with DGBI. We hypothesized that our support system could distinguish patients with DGBI, particularly those with FD, from healthy controls, with sufficiently high accuracy above chance level.

## Materials and methods

This prospective study was conducted from August 2022 to March 2023 at the Kawasaki Medical School General Medical Center and Kawasaki University of Medical Welfare. This study protocol followed the tenets of the Declaration of Helsinki and was approved by the Research Ethics Committee of Kawasaki Medical School Hospital (IRB No. 5696-01). Written informed consent was obtained from all participants, and we followed the Society for Functional Near-Infrared Spectroscopy guidelines for fNIRS studies [17].

This study’s primary endpoint was to establish an AI-based diagnostic model to distinguish between patients with DGBIs and healthy controls, those with FD and healthy controls, those with IBS and healthy controls, and patients with FD and those with IBS.

We initially set the sample size as 12 participants in each of the three groups, according to previous studies that evaluated emotional processing using fNIRS [18,19]. A post-hoc power analysis was conducted to confirm the study power after obtaining the data.

Patients and healthy controls

We included outpatients with FD and IBS, subtyped according to the ROME IV criteria, at Kawasaki Medical School General Medical Center [1,2]. In this study, patients with FD or IBS were considered to have DGBIs, whereas healthy controls were participants without GI symptoms or a current medical history of GI disorders, such as gastroesophageal reflux disease, inflammatory bowel disease, and GI cancers. The inclusion criteria were as follows: age >20 and <65 years, either sex, and willingness to participate in this study. The exclusion criteria were participants with active Helicobacter pylori infection, malignant disease, iron deficiency anemia, and a history of abdominal surgery. Blood tests, including complete blood count and C-reactive protein and blood electrolyte level assessments, were performed for all patients, and we performed upper GI endoscopy in all patients with FD and colonoscopy or abdominal computed tomography in those with IBS to evaluate organic disorders.

Food images and preference rating

**Figure 1:**
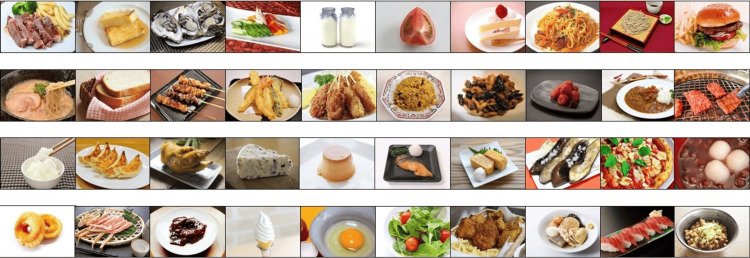
Food images used in this study. Forty different images including 10 fatty food and 10 light food images were selected.

Forty different food images, including images of fatty and light foods, were used (Figure 1). Overall, we asked 40 healthy participants without GI symptoms or active GI disorders to rate the grades of food fattiness using a visual analog scale (VAS). Food images that ranked in the top 10 and those with at least 20 g of fat per 100 g were defined as fatty foods. In contrast, food images ranked in the last 10 and those containing at most 4 g of fat per 100 g were defined as light foods.

Preference for food images, displayed in the center of a laptop computer screen, was assessed by all participants using VAS, with ratings ranging from not preferable (1) to preferable (100).

Measurements and analysis of fNIRS

**Figure 2:**
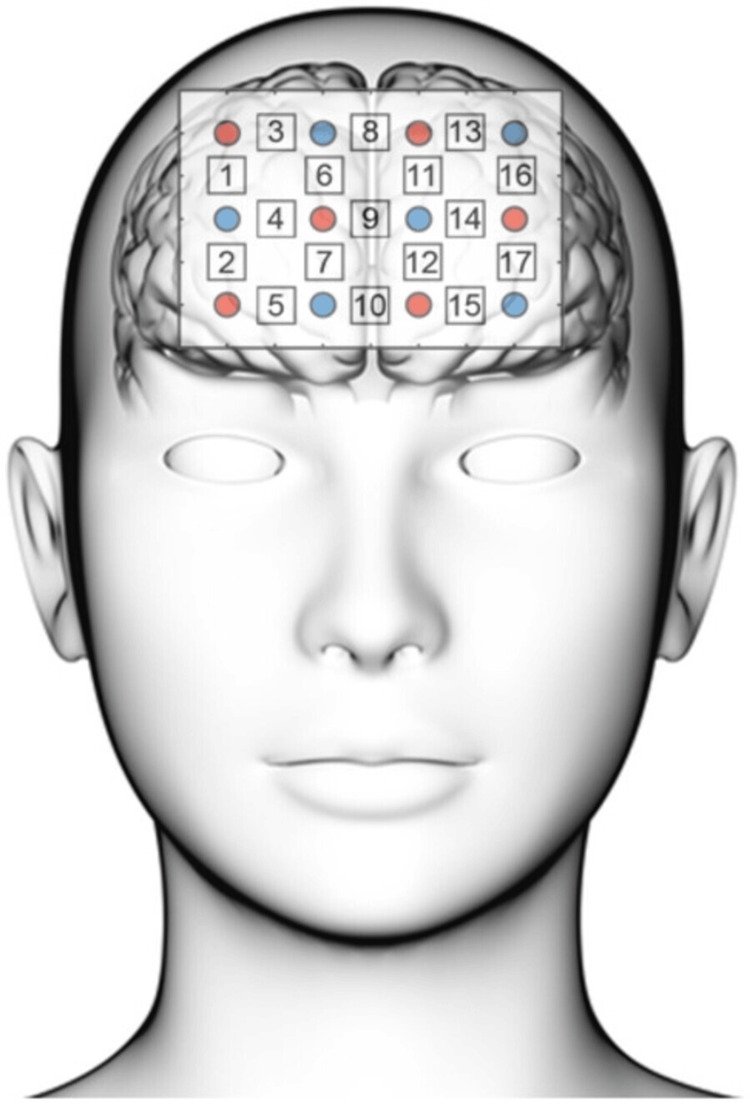
Orientation of the fNIRS probes. The red and blue circles show the emitter and detector probes, respectively. The numbers represent the channel number. fNIRS: functional near-infrared spectroscopy

We evaluated oxygenation changes in the PFC using Spectratech OEG-17APD (Spectratech Inc., Yokohama, Japan) at two wavelengths (770 and 840 nm) based on the modified Beer-Lambert law [20]. We attached and fixed the fNIRS probes to the forefront according to the 10-10 electrode placement system, with the lowest probe line located on Fpz (Figure 2). Overall, 17 channels were evaluated in this study using a 3×4 probe arrangement with a 3-cm inter-optode distance.

Experiment and data analysis of fNIRS

The experimental procedure was similar to that described in a previous study [6]. Briefly, the participants were instructed to sit on a chair in a quiet room facing a laptop computer. After evaluating the baseline data, each food image was randomly displayed for 7 s, followed by a 10 s inter-trial interval for a total of 40 food images. The brain activity was recorded using fNIRS, while participants viewed the images. We adopted the PsychoPy software version 2022.1.3 (Open Science Tools Ltd, United Kingdom) to perform the procedure [21]. After the experiment, Z-scores were obtained from the fNIRS raw data following the procedure described in a previous study [6].

Establishing the diagnostic models

Binary classifications were used to distinguish between the DGBI and healthy control, IBS and healthy control, FD and healthy control, and FD and IBS groups. An artificial neural network model provided by the Statistics and Machine Learning Toolbox (MathWorks Inc., Natick, MA, USA) was used as the classification method. The function “fitcnet” has been used in previous clinical studies [22,23], and we set up a single intermediate layer with 64 nodes.

**Figure 3:**
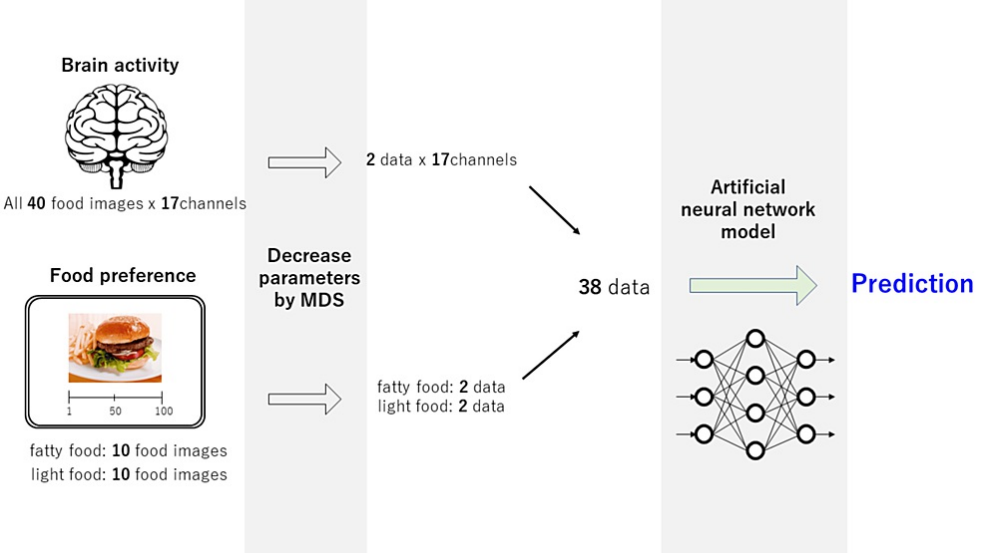
Flow of AI-supported diagnosis prediction. Raw data obtained from fNIRS are processed through MDS. Compressed data are used to predict diagnosis using an artificial neural network model. AI: artificial intelligence; fNIRS: functional near-infrared spectroscopy; MDS: multidimensional scaling

To prevent overfitting, the parameters were reduced using multidimensional scaling (MDS). Finally, two brain activity parameters from 40 parameters (each mean z-score of food images) and two food preference parameters from 10 parameters (each VAS score of fatty and light food images) were obtained for each participant. Overall, we used 38 parameters for the AI model [2 parameters × 17 channels for brain activity and 2 parameters × 2 food types (fatty and light foods) for food preference]. The flow of the data processing for AI prediction is shown in Figure 3. Furthermore, the training and test data ratio was 8:2, and we calculated the classification results using 10 training and test dataset combinations for sufficient cross-validation.

Statistical analysis

We used the Student’s t-test to compare continuous variables between two groups such as healthy controls and patients with DGBI, and the χ2 test was used to compare the frequencies between two groups (healthy controls and patients with DGBI) and among three groups such as healthy controls, patients with FD, and those with IBS. Comparisons among three groups (healthy controls, FD, IBS) with respect to continuous data, such as age and disease duration, were conducted using analysis of variance with the Tukey-Kramer post-hoc test. All calculations were performed using MATLAB (MathWorks Inc., Natick, MA, USA), and two-sided values of p<0.05 were considered statistically significant.

## Results

Clinical characteristics

Overall, 41 patients, including 12 with FD, 13 with IBS, and 16 healthy controls were enrolled in the study. Table 1 presents the clinical characteristics, including age, sex, body mass index (BMI, calculated by dividing weight in kilograms by height in meters squared), and disease duration, of the patients with DGBI and healthy controls. Among the 12 patients with FD, the number of those with epigastric pain syndrome (EPS), postprandial distress syndrome (PDS), and both EPS and PDS were four, six, and two, respectively. Among the 13 patients with IBS, the number of those with constipation-predominant IBS (IBS-C), diarrhea-predominant IBS (IBS-D), and mixed IBS (IBS-M) were four, six, and three, respectively. Table 2 shows a comparison among healthy controls, patients with FD, and those with IBS regarding demographic data and subtypes of FD and IBS. Clinical characteristics including age, sex, BMI, alcohol intake and smoking status were not statistically different among groups.

**Table 1:** Clinical background of healthy controls and patients with DGBI DGBI: disorder of gut-brain interaction, SD: standard deviation; n: number; BMI: body mass index p-values were calculated using Student’s t-test^a^ and chi-square test^b^

Variables	Control (n=16)	DGBI (n=25)	p-values
Age (mean [SD], years)	43.6 (3.3)	38.7 (2.6)	0.367^a^
Male (n [%])	5 (31.3)	8 (32.0)	0.959^b^
BMI (mean [SD], kg/m^2^)	22.8 (0.8)	22.3 (0.7)	0.667^a^
Alcohol: current drinker (n [%])	6 (37.5)	7 (28.0)	0.825^b^
Smoking (n [%])	0 (0)	0 (0)	(-)
Food allergy (n [%])	3 (18.7)	7 (28.0)	0.508^b^
Disease duration (mean [SD], years)	0 (0)	7.88 (6.3)	(-)

**Table 2:** Clinical background of healthy controls and patients with FD and those with IBS FD: functional dyspepsia; IBS: irritable bowel syndrome; SD: standard deviation; n: number; BMI: body mass index; EPS: epigastric pain syndrome; PDS: postprandial distress syndrome; IBS-C: constipation-predominant IBS; IBS-D: diarrhea-predominant IBS; IBS-M: mixed IBS p-values were calculated using analysis of variance^a^, chi-square test^b^, and Student’s t-test between FD and IBS^c^

Variables	Control (n=16)	FD (n=12)	IBS (n=13)	p-values
Age (mean [SD], years)	43.6 (3.3)	36.5 (3.8)	40.7 (3.6)	0.418^a^
Male (n [%])	5 (31.3)	4 (33.3)	4 (30.8)	0.965^b^
BMI (mean [SD], kg/m^2^)	22.8 (0.8)	21.2 (1.0)	23.2 (0.9)	0.362^a^
Alcohol: current drinker (n [%])	5 (31.3)	3 (25.0)	4 (30.8)	0.904^b^
Smoking (n [%])	0 (0)	0 (0)	0 (0)	(-)
Disease duration (mean [SD], years)	0 (0)	6.83 (4.6)	10.07 (7.0)	0.213^c^
Subtypes (n [%])				
EPS	(-)	4 (33.3)	(-)	(-)
PDS	(-)	5 (41.7)	(-)	(-)
Mixed	(-)	2(16.7)	(-)	(-)
IBS-C	(-)	(-)	4 (30.7)	(-)
IBS-D	(-)	(-)	6 (46.2)	(-)
IBS-M	(-)	(-)	3 (23.1)	(-)

Diagnostic model

The dataset obtained in our previous study was used in this pilot trial [6]. We used 38 parameters for each participant after dimensionality reduction to differentiate between the two groups. Table 3 presents the classification results for each pair. The sensitivity, specificity, positive predictive value, negative predictive value, and accuracy of the AI-based diagnostic model for differentiating patients with DGBI from healthy controls were 79.2%, 55.6%, 80.6%, 53.4%, and 72.3%, respectively. Overall, the accuracy of each classification was higher than the chance level. Among the comparisons, the classification between the healthy control and FD groups showed the highest accuracy (77.1%), whereas that between the FD and IBS groups had the lowest accuracy (60.3%).

**Table 3:** AI-based classification parameters AI: artificial intelligence; DGBI: disorder of gut-brain interaction; FD: functional dyspepsia; IBS: irritable bowel syndrome; SD: standard deviation

Pairing % (SD)	Sensitivity	Specificity	Positive predictive value	Negative predictive value	Accuracy
Healthy control vs. patients with DGBI	79.53 (8.2)	55.67 (15.6)	80.69 (13.1)	53.44 (20.7)	72.39 (6.0)
Healthy control vs. patients with FD	73.43 (16.7)	81.25 (13.5)	73.45 (16.2)	79.20 (17.0)	77.19 (3.5)
Healthy control vs. patients with IBS	67.92 (25.5)	72.29 (13.5)	60.66 (27.3)	74.83 (23.1)	63.54 (7.6)
Patients with FD vs. those with IBS	60.82 (18.4)	57.07 (13.1)	63.47 (18.3)	54.88 (12.6)	60.30 (7.4)

## Discussion

In this study, we developed a diagnostic system based on brain activity information and food preference using neural network machine learning. The accuracy was highest in the distinction between patients with FD and healthy controls and lowest in the distinction between patients with FD and those with IBS. The results are consistent with those in our previous study, in which we found significant differences in food preference and brain activity between patients with FD and healthy controls, whereas mild differences were observed between patients with IBS and healthy controls and between patients with IBS and FD [6]. The accuracy of distinguishing patients with FD from healthy controls is clinically relevant. Several AI-based models previously reported for diagnosing IBS and FD have an accuracy of approximately 80% [14]. Our classification model is thus acceptable for integrating clinical conditions, considering its 77% accuracy in distinguishing patients with FD from healthy controls and the procedure’s non-invasiveness and ease of use. Regarding the classification systems using brain activity, models using fMRI data differentiated patients with FD from healthy controls with 88% accuracy [24], and patients with IBS from healthy controls with 70% accuracy [25]. We constructed a model using only brain activity data from the PFC. Nevertheless, our model showed an accuracy similar to those in previous fMRI studies using whole-brain data, indicating that the model can be a useful alternative in clinical settings given the ease of use of fNIRS.

The accuracy of the model to distinguish patients with FD from those with IBS was much lower (60%) than that to distinguish healthy controls from patients with FD (77%). This result suggests that FD and IBS share similar brain activity patterns but can have slightly different brain function-related aspects. Although distinguishing FD from IBS is clinically simple, with or without impairment of bowel movement, the difference in pathophysiology between the two disorders has rarely been investigated. We initially demonstrated the differences in brain activity between patients with FD and those with IBS in similar tasks [6]; however, the difference was smaller than that between patients with FD and healthy controls. Overlap between FD and IBS has been frequently reported [26,27]. These real-world data and our results suggest that FD and IBS have similar pathophysiology; however, they can show specific brain function patterns depending on the task. Therefore, tasks that induce large differences in brain activity among these groups are required to establish a more useful system to distinguish patients with IBS from healthy controls and those with FD.

This study had some limitations. First, this study had a selection bias due to its single-center nature, and all participants were Japanese. Since we enrolled patients consecutively, those with DGBI had different backgrounds, such as treatment, disease duration, and comorbidities. Regarding comorbidities, we confirmed no participants with psychiatric illness were enrolled, but other metabolic disorders such as diabetes and hyperlipidemia which can influence food preference were not fully investigated. Additionally, we aimed to provide preliminary data in this pilot study. Therefore, international large-scale studies should be conducted at multiple centers. Second, a limited dataset of brain activity was used in this study. fNIRS can only evaluate superficial brain activity, and our data were obtained only from the PFC. However, the accuracy of these data is sufficiently precise. Moreover, fNIRS is superior because of its better time resolution, lower running cost, and shorter preparation period than other modalities, including fMRI and positron emission tomography (PET). Furthermore, this noninvasive technique using portable equipment can be performed in a normal posture and requires only minimal restraint without excluding individuals with non-removable metal objects in their bodies or tattoos. These characteristics indicate that fNIRS is more suitable than fMRI and PET for evaluating participants’ brain activity in daily clinical settings. Third, our model cannot provide sufficient accuracy to distinguish patients with IBS from healthy controls and those with FD, perhaps because tasks using food images cannot induce specific brain activity in patients with IBS. One possible reason was that pictures instead of the real food were used. In addition to tasks related to food, several tasks including rectal distention and the Wisconsin Card Sorting Test were confirmed to induce different brain activity in patients with IBS compared to healthy controls. Therefore, other tasks should be employed to detect differences in brain activity in order to distinguish patients with IBS from healthy controls such as rectal distention and cognitive tasks that have been used in previous studies and have yielded significant differences in brain activity [8,28]. Accordingly, further data are required to establish a more useful model for IBS diagnoses.

## Conclusions

DGBI are any disorder where no organic clinical abnormalities are detected which makes them difficult to accurately diagnose. This diagnostic model had adequate accuracy, particularly in patients with FD, and could distinguish patients with DGBI from healthy controls with the highest accuracy in the distinction between patients with FD and healthy controls. This model can help clinicians when facing patients who cannot sufficiently express their complaints or whose complaints are not reliable. This pilot trial provides an initial step toward establishing an AI-supported diagnostic system using objective data on brain activity and food preference. Our findings also indicate that fNIRS data can support objective diagnosis and assessment in daily clinical settings which will decrease the time to diagnosis.
